# Contributions to Unsupervised Online Misalignment Detection and Bumper Error Compensation for Automotive Radar †

**DOI:** 10.3390/s23156785

**Published:** 2023-07-29

**Authors:** Alexandru Bobaru, Corina Nafornita, George Copacean, Vladimir Cristian Vesa, Michael Skutek

**Affiliations:** 1 Communications Department, Politehnica University of Timisoara, 300223 Timisoara, Romania; corina.nafornita@upt.ro; 2 Hella Romania, 300011 Timisoara, Romania; george.copacean@forvia.com (G.C.); vladimircristian.vesa@forvia.com (V.C.V.); 3 Hella GmbH & Co. KGaA, 59552 Lippstadt, Germany; michael.skutek@forvia.com

**Keywords:** automotive radar, horizontal misalignment, vertical misalignment, azimuth calibration, elevation calibration, bumper compensation, automatic calibration, ADAS, automated driving, AD

## Abstract

One of the fundamental sensors utilized in the Advanced Driver Assist System (ADAS) is the radar sensor. Automotive-related functions need highly precise detection and range of traffic and surroundings; otherwise, the whole ADAS performance suffers. The radar placement beneath a bumper or a cover, the age or exposure to accidents or vehicle vibration, vehicle integration, and mounting tolerances will impact the angular performance of the radar sensor. In this research, we present an unsupervised online method for elevation mounting angle error compensation and a method for bumper and environmental error compensation in the azimuth direction. The proposed methods need no specific calibration jig and may be used to replace traditional initial calibration methods; they also enable ongoing calibration throughout the sensor’s lifespan. A first proposed standalone method for vertical alignment uses stationary radar targets reflected from the environment to calculate a vertical misalignment angle with a line-fitting algorithm. The vertical mounting error compensation approach delivers two types of correction values: a dynamic value that converges quickly in the case of minor accidents and a more stable correction value that converges slowly but offers a long-term compensation value over the sensor’s lifespan. A second proposed solution uses the vehicle velocity and radar targets properties, like relative velocity and measured azimuth angle, to calculate an individual azimuth correction curve. Real-world data collected from drive testing with a 77 GHz series automobile radar was used to analyze the performance of the proposed methods, yielding encouraging results.

## 1. Introduction

Accurate environmental perception by the plethora of sensors that make up Automated Driving (AD) systems is their most difficult problem. One such sensor is the automotive radar. Due to active monitoring and risk assessment in the context of providing warnings for the driver, the radar offers active functionalities like Environment Detection, Cross-Traffic Alert, and Lane Change Assist that require precise detection and ranging of the traffic and environment. The detection of radar targets, objects, and environments can be compromised by a problem with the sensor alignment, which also affects the system’s overall performance and perception accuracy. Errors in the azimuth directions can lead to the wrong lane assignment of the tracked objects, while mounting elevation errors or the overall error caused by heavy cargo in the trunk, potholes, or even minor accidents can lead to the wrong classification of the objects or environmental detection errors. Angular self-calibration of sensors is a very important topic in the automotive field due to the negative impact on functionalities in its absence.

There are offline calibration methods, which are carried out in the automotive customer’s production plant or in service (End-of-Line Calibration). These involve placing the vehicle in a well-determined set-up together with various reference objects, whose attributes such as position, azimuth, and elevation angles are well known, and respecting strict tolerances [1,2,3]. These methods are disadvantageous both financially and in terms of the time required to perform the calibration of each vehicle.

Other methods of calibrating mounting angles in azimuth and elevation can also be obtained in a controlled environment, but with fewer restrictions or using data from other automotive systems, such as Lidar and cameras [4,5,6,7,8,9]. The main advantage of these methods is that they provide an initial elevation mounting angle calibration if the radar does not support elevation angle measurement. However, a big disadvantage of these methods is that they only provide an initial calibration of the mounting angles and do not provide long-term compensation.

Online azimuth mounting error compensation methods can be based on a reference consisting of a static object parallel to the driving direction of the vehicle (street or highway edges) [10,11] or on data fusion from more sensors [12,13].

In [14], the method used calculates the errors in azimuth and elevation based on the vehicle speed, sideslip angle, and radar target angle and speed, respectively. The misalignment angle is then calculated either using a recursive filter or by batch processing. A procedure is proposed in [15] where the mounting angles for azimuth and elevation are calculated indirectly, without knowing the radar position, thus returning a sensor position correction matrix, considering the Cartesian coordinate system. In [16], we proposed an azimuth autocalibration method based on the corrected ego velocity estimation and the measured azimuth and relative velocity of stationary targets that was able to calculate a fast and slow angle while minimizing the effect of the bumper errors over the mounting azimuth angle correction calculation.

For the compensation of local angular distortions in azimuth, there are different offline methods based either on simulations that include knowledge of the bumper properties or on methods that require receiving a signal from a target with a known angle [17].

For automotive radars, these types of offline calibration present great difficulties due to the need to calibrate the sensors after mounting them behind the bumper as well as the cost related to allocating a special space for the setup. These types of corrections should ideally be automatic, taking into account both radar wear and the wear and tear of bumper properties over time. M. Harter et al. [18] developed an automatic method to compensate local offsets for MIMO (Multiple Input and Multiple Output) radars, but this requires a special setup and presents difficulties in adapting to SIMO (Single Input and Multiple Output) radars. K. Suzuki et al. [19] proposed a feasible method for SIMO radars that generates a correction table by performing a regression of the displacement error (bias) function. A disadvantage of this solution is the high computing power required by using the Markov Chain Monte Carlo (MCMC) method for performing the regression of the error function. Another method of online correction of local angular distortions in azimuth [20] consists of estimating the real trajectory (reference) of an object that performs a maneuver to overtake its own vehicle. During the overtaking maneuver, the object will be tracked, and at each instance, the targets around the object will be used in the calculation of an average distance and average measured angle. After estimating the ideal trajectory, differences in distances between the reference position of the object and the position of each instance will be observed, which will lead to the calculation of angle differences.

In [21], we proposed an angular bumper error compensation method that was based on stationary references (e.g., road edge, metal guardrail) and the lateral position of the stationary targets around these references in order to calculate the needed angular azimuth correction. The resultant individual correction was used to create and update an angular correction characteristic. 

Our goal is to propose online unsupervised azimuth and elevation mounting error correction methods as well as an online unsupervised azimuth bumper error compensation method. The following are the paper’s main contributions:

- A fully functional stand-alone model capable of adapting the radar system to elevation mounting angle errors and serving as both an initial and ongoing calibration [22].
- Two separate sets of parameter combinations lead to the use of two correction values: robust and dynamic. The dynamic value will be utilized to identify rapid changes, such as in the case of accidents, while the robust value will reflect the stable, overall global compensation angle. Which value will be utilized to correct the radar raw targets will be determined based on a hysteresis mechanism [22].
- An improved angular bumper error compensation version of the method from [21] that delivers continuous adjustments based on stationary targets and host velocity, followed by the implementation of a regression model and a smoothing approach to assure the model’s stability and accuracy.

The paper is structured as follows: The elevation autocalibration method is presented in Section 2. The bumper error compensation method is presented in Section 3, and the numerical results and discussions are shown in Section 4. In Section 5, we explain both the superiority and the shortcomings of the proposed methods. We provide conclusions and some potential future study directions in Section 6.

## 2. Unsupervised Online Vertical Misalignment Detection Algorithm for Automotive Radar

**Figure 1:**
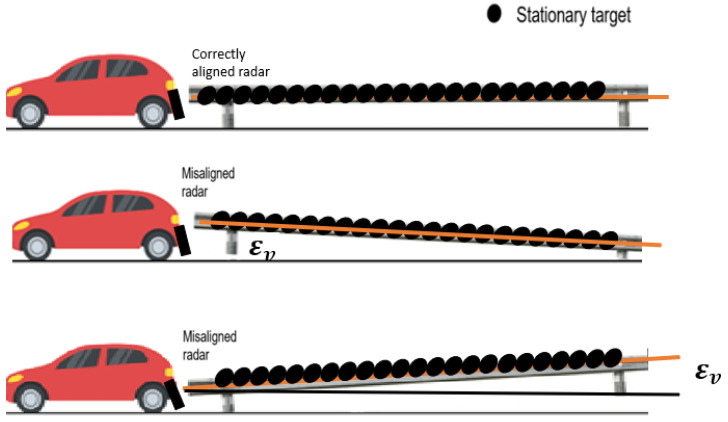
Scenario for the estimation of the vertical correction for no elevation mounting error (**first picture**), for negative elevation mounting error (**second picture**), and for positive elevation mounting error (**third picture**).

In the case shown in Figure 1, the car is traveling straight while in an appropriate environment (such as a highway), and radar reflections from stationary objects (such as a metal guardrail) show a vertical inaccuracy ($\varepsilon_{v}$) that is the result of the radar sensor’s improper elevation mounting.

### 2.1. The Elevation Automatic Calibration Model

**Figure 2:**
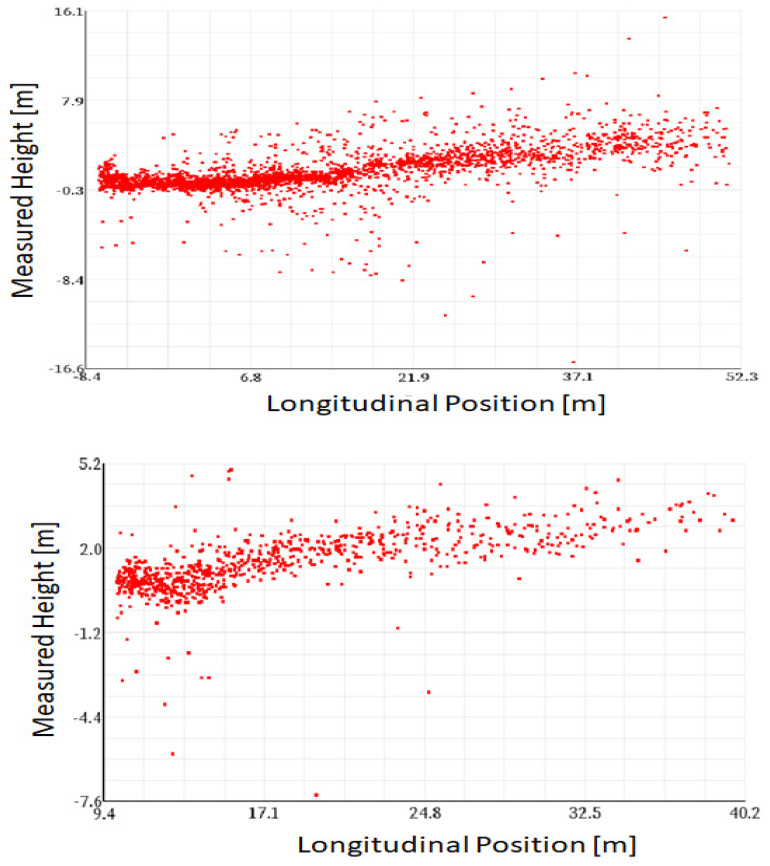
Example of all targets from multiple consecutive radar cycles (**upper picture**) compared to the suitable targets used by the algorithm (**lower picture**) in the context of a misaligned radar in the elevation direction.

For the calculation of the elevation misalignment angle, only suitable targets will be used. Selection factors such as raw target stationarity, longitudinal and lateral position, target height, angular elevation interval, signal-to-noise ratio (SNR), and others are used to determine the suitability of a target. The benefit of rigorous target selection can be observed in Figure 2.

These targets can be used in a line-fitting method like least squares regression, based on target longitudinal position and height, calculated in Equations (1) and (2), to obtain an offset and a slope, then trigonometrically calculating the angle based on the slope of the resulted line:

(1) $$\tilde{z}=\tilde{R}\cdot \sin\left(\beta +\varepsilon_{\beta }+\eta_{\beta }\right)$$

(2) $$\tilde{x}=\tilde{R}\cdot \cos\left(\alpha \right)$$

where $\tilde{z}$ and $\tilde{x}$ are the measured height and longitudinal position of the target, respectively; $\tilde{R}$ is the measured distance of the target; *α* is the azimuth angle; β is the elevation angle; $\varepsilon_{\beta }$ is the vertical mounting error; and $\eta_{\beta }$ is the Gaussian noise.

However, storing and executing the least squares regression on that many pairs of longitudinal positions and heights can be too computationally demanding in an automotive system, where memory and runtime requirements are very tight. 

**Figure 3:**
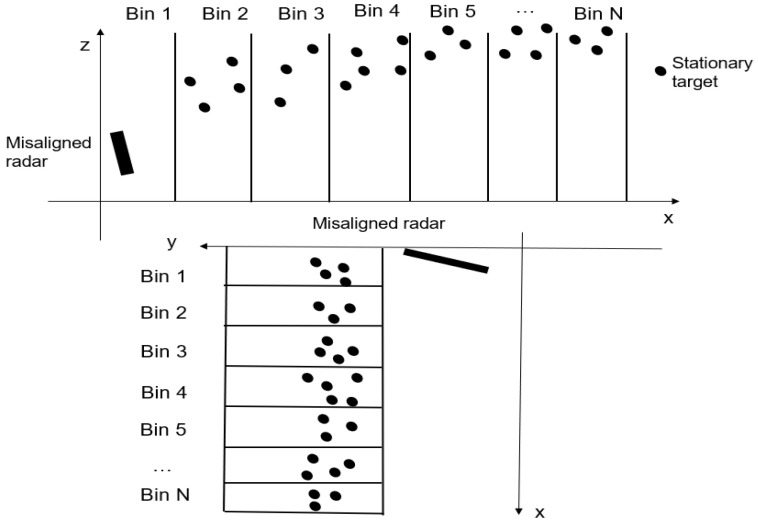
Example of target association to the longitudinal bin as seen from lateral perspective (**upper picture**) and birdview perspective (**lower picture**) [22].

Our proposed method indicates the splitting of the longitudinal axis into equidistant bins, as seen in Figure 3, and each target will be attributed to its bin according to Equation (3):

(3) $$i=\left[\;\frac{\tilde{x}-x_{start}}{x_{step}}\right],\;x_{start}<\tilde{x}<x_{end}\;,$$

where *i* is the height bin to which the target is allocated to $x_{start}$, and $x_{end}$ are the minimum and maximum longitudinal positions allowed from the target selection phase, respectively, while $x_{step}$ represents the bin granularity in the longitudinal direction.

Each bin is represented by the number of used targets, k, and a mean height calculated as in [23] with the filtering factor *f*, as seen in Equation (4):

(4) $${\bar{z}}_{i\_current}={\bar{z}}_{i\_previous}+f\cdot \left(\tilde{z}-{\bar{z}}_{i\_previous}\right)$$

When a minimum number of height bins ($Min_{Bins}$) accumulated at least $Min_{targets}$ targets each, a least squares regression process [24] will be applied on the height bins, resulting in a slope *m* and intercept *b*, presented in Equations (5) and (6):

(5) $$m=\frac{N\sum \left(i{\bar{z}}_{i}\right)-\sum i\sum {\bar{z}}_{i}}{N\sum \left(i^{2}\right)-\sum {\bar{z}}_{i}{}^{2}}$$

(6) $$b=\frac{\sum {\bar{z}}_{i}-m\sum i}{N}$$

where *N* is the number of accepted height bins.

One instance of elevation correction Δβ is calculated as follows:

(7) $$\tilde{\Delta \beta }=-\tan^{-1}\left(m\right)$$

It is necessary to use a filtering approach to produce an accurate estimation. In this paper, we propose to use an exponential moving average with the factor *g*:

(8) $$\Delta \beta =\Delta \beta_{previous}+g\cdot \left(\tilde{\Delta \beta }-\Delta \beta_{previous}\right)$$

However, a correction $\tilde{\Delta \beta }$ will only be used in the filtering process if the root mean square error (rmse) from the statistics that led to the correction calculation is small enough. If the process is successful, the new value can be used to correct the raw targets, then the bin statistics will be reset, and the process will restart.

### 2.2. Purpose-Based Correction

It is essential to have a stable calibration value that can offer long-term electronic compensation for sensor misalignment during the sensor’s lifespan. The algorithm should employ a slower adaptation in order to produce a steady and accurate calibration value. It is also crucial for the algorithm to quickly converge to the new compensation value in critical scenarios, such as accidents where the bumper is damaged and causes a sensor misalignment. For this, it is recommended to use a more rapid and dynamic adaptation while anticipating a somewhat lower accuracy than the robust filtering method.

We propose computing the robust and dynamic angles using two separate sets of parameters in order to achieve the aforementioned objectives for the following: the longitudinal step for a height bin, the number of required bins, the minimum number of required targets for each bin, and the filter factor for angle calculation limit for a successful usage of a calculated correction value. 

For the robust angle correction $\Delta \beta_{r}$, we employ a higher number of necessary bins and necessary targets for each bin, a lower filtering factor, and a higher value of the rmse limit than for the dynamic angle correction $\Delta \beta_{d}$. 

The correction value $\Delta \beta_{u}$ that will be used for the target correction and misalignment detection is described in (9):

(9) $$\Delta \beta_{u}=\left\{\begin{array}{l} \Delta \beta_{r},\;if\;c<h_{min}\\ \Delta \beta_{d},\;if\;c>h_{max}\\ previous\;state\;used,\;if\;c\in \left[h_{min},h_{max}\;\right] \end{array}\right.$$

where $c=\left|\Delta \beta_{r}-\Delta \beta_{d}\right|$, and $h_{min}$, $\;h_{max}$ are the minimum and maximum required absolute differences for the hysteresis trigger, respectively.

## 3. Unsupervised Online Azimuth Bumper Error Compensation Algorithm for Automotive Radar

In [21], we proposed a method for compensating the azimuth angle bumper errors. Given a radar cycle *k*, the created raw target list, containing stationary targets, will be filtered by different suitability criteria, and the targets that pass all the conditions are to be used in an Azimuth Angle Correction Block. Over multiple radar cycles, a plausible Internal Angle Correction Characteristic (IACC) is generated using least squares regression and exponential moving average processes. The IACC’s corrections will undergo a smoothing process, generating a smoothed angle characteristic that will be the basis for the SACC (System Angle Correction Characteristic) calculation. The new SACC is then used by the Radar Signal Processing block in the next radar cycle. The diagram of the local offset compensation procedure is shown in [21].

The correction calculation in the last method [21] was performed using the estimated road edge (e.g., metal guardrail) position and the measured lateral position of the raw targets, under the assumption that the selected targets belong on that specific road edge; thus, the difference in the lateral positions would provide an angular correction $\alpha_{diff}$ and an assignment angle $\alpha_{assignment}$. The pair is then fed to the IACC update block, which has a table-like form with a starting angle, number of points, and granularity. The nearest left and right neighbors of the pair are found, and the corrections of these neighboring points are calculated from $\alpha_{diff}$ via a linear least squares fitting method. After a number of such IACC updates have taken place, a validation process takes place for the IACC where every supporting point is smoothed based on its neighboring points while also performing ambiguity avoidance, generating the SACC. The method performs reasonably well in the lower-valued angles while showing a lack of accurate corrections at the edges of the angular interval due to a lack of suitable targets around the field of view of the sensor.

Our new method adds improvements to the previous process by changing the way the correction calculation is performed in such a way that it can correct the whole azimuth angular interval while maintaining the same accuracy and by using an improved assignment angle calculation, regression model, and quality measure.

### 3.1. Correction Calculation

In [16], we defined the measured azimuth angle $\tilde{\alpha }$ as a function of the ideal angle *α*, horizontal mounting error $\varepsilon_{h}$, systematic individual error (bumper related error) $\varepsilon_{sys}\left(\;\alpha \right)$ and noise $\eta_{\alpha }$:

(10) $$\tilde{\alpha }=\alpha +\varepsilon_{h}+\varepsilon_{sys}\left(\alpha \right)+\eta_{\alpha }$$

Based on Equations (1) and (2) from [16], the relationship between the corrected and odometer error-free vehicle velocity $v_{ego}$, the relative velocity $\tilde{v_{r}}$ of a stationary target, and its reference azimuth angle *α*, without considering the elevation angle, has the following form:

(11) $$\tilde{v_{r}}≅-v_{ego}\cdot \;\cos\left(\alpha \right)$$

(12) $$\alpha =arccos\left(\frac{\tilde{v_{r}}}{v_{ego}}\right)$$

(13) $$\varepsilon_{h}+\varepsilon_{sys}\left(\;\alpha \right)+\eta_{\alpha }=\tilde{\alpha }-arccos\left(\frac{\tilde{v_{r}}}{v_{ego}}\right)\;$$

If we consider the odometry errors of the velocity due to systematic errors like a deviating tire circumference, then $v_{ego}$ can be rewritten depending on a factor ρ and Gaussian noise $\eta_{v_{ego}}$ as follows:

(14) $$\tilde{v_{ego}}=\rho \cdot v_{ego}+\eta_{v_{ego}}\;$$

In addition, the disturbance also influences the measurement of the radial velocity $\tilde{v_{r}}$ in such a way that it can also be described as Gaussian noise $\eta_{v_{r}}$:

(15) $$\tilde{v_{r}}=v_{r}+\eta_{v_{r}}$$

Assuming that the targets originate in the azimuth plane, then:

(16) $$\rho \cdot \frac{\tilde{v_{r}}-\eta_{v_{r}}}{\tilde{v_{ego}}-\rho \cdot \eta_{v_{ego}}}=\cos\left(\tilde{\alpha }-\varepsilon_{h}-\varepsilon_{sys}\left(\;\alpha \right)-\eta_{\alpha }\right)$$

Neglecting the noise $\eta_{v_{ego}}$ from the intrinsic velocity measurement results in:

(17) $$\frac{\tilde{v_{r}}}{v_{ego}}-\frac{\eta_{v_{r}}}{\tilde{v_{ego}}}=\frac{1}{\rho }\cdot \cos\left(\tilde{\alpha }-\varepsilon_{h}-\varepsilon_{sys}\left(\;\alpha \right)-\eta_{\alpha }\right)\;$$

Considering that $v_{ego}$ has been compensated and is free of errors, as well as the mounting angle error $\varepsilon_{h}=0$, the SACC calculation process can start. In [16], it has been found that in principle, an offset determination, according to (13), in fixed angular ranges corresponds to the determination of the corresponding distortions. Another approach, similar to [21], is a regression of the data points (*x*, *y*) = ($\tilde{\alpha },\frac{\tilde{v_{r}}}{v_{ego}}$). 

For the purpose of this section, the factor ρ will be assumed to be ρ=1. The approaches of having fixed measurement angles and fixed estimation angles will be studied, and then a general approach will be presented that will allow for the consideration of the noise terms in the *x* and *y* components. In the end, the pair of ($\alpha_{assignment},\;\alpha_{diff}$) will be calculated and sent to the IACC update block. In the first approach, $\alpha_{assignment}=\tilde{\alpha }$. 

The problem with the first approach is that $\tilde{\alpha }$ is modeled with $r_{a}=e_{a}+\eta_{\alpha }$, where $e_{a}$ is a DC component of the noise that describes the way in which the targets from other angular ranges are measured in the considered measurement angular range. This component is not generally free of mean values for a given $\tilde{\alpha }$. If, for example, we consider the area behind a vehicle (very low angles) when driving along a barrier, the majority of the detections in these angular ranges will be traced back to the barrier reflection, resulting in a one-sided noise process. This approach is heavily influenced by the measurement angle noise, assuming very large standard deviations of the corrections depending on the angular range and directional characteristics of the antennas. This means that the pair $\left(\alpha_{assignment},\;\alpha_{diff}\right)$, where $\alpha_{assignment}=\tilde{\alpha }+\Delta \alpha$ is not a favorable approach as input for the regression function of IACC. In this case, Δα is the mounting angle correction is applied over the measured angle in such a way that SACC will strictly calculate the bumper compensation errors. 

The second approach implies the combination of the same y-values $\frac{\tilde{v_{r}}}{v_{ego}}$ into positive and negative angular ranges and forming the expected value of the associated x-values. Since y-fixed values are grouped separately according to positive and negative values, then the estimated angle is $\hat{\alpha }=sign\left(\tilde{\alpha }\right)\cdot arccos\left(\frac{\tilde{v_{r}}}{v_{ego}}\right)$. However, wrong decisions in the sign assignment can occur, which in turn will result in systematic distortions $e_{a}$ in the angle noise for a given estimate $\hat{\alpha }$ since the targets are assigned to an estimation angle only from the positive or only from the negative angle range, leading to a one-sided noise process in the very small angle ranges. The distortion $e_{a}$ will have a particularly strong effect in small angular ranges, which is fatal for functionalities like Lane Change Assist or Autopilot. 

To take into account the size ratio of the noise terms, the y-axis is scaled accordingly with a scale factor γ, which results from the ratio of the noise terms of the x and y components. Since the function is generally unknown but should show a cosine characteristic, we can use it as the basis for the formation of the expected value. After appropriate scaling, orthogonal straight lines n($x_{i},\;x)$ to the cosine function are formed in equidistant angular steps [22]. After that, the cosine and the straight lines are scaled back again, resulting in straight lines m($x_{i},\;x)$, where $s_{i}=\frac{1}{\gamma^{2}\cdot \sin\left(x-x_{i}\right)}$:

(18) $$m\left(x_{i},\;x\right)=\cos\left(x_{i}\right)+s_{i}\cdot \left(x-x_{i}\right)$$

**Figure 4:**
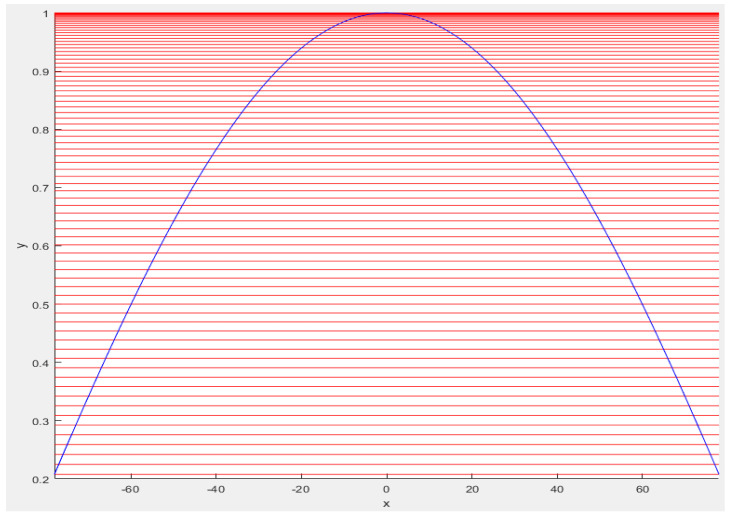
Direction of expected value formation for γ → ∞. The red lines represent the direction of the expected value formation, while the blue line represents an ideal cosine.

**Figure 5:**
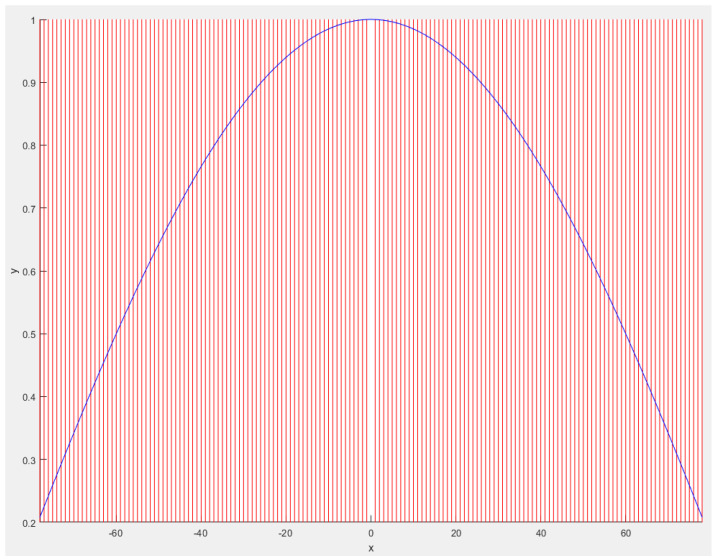
Direction of expected value formation for γ → 0. The red lines represent the direction of the expected value formation, while the blue line represents an ideal cosine.

**Figure 6:**
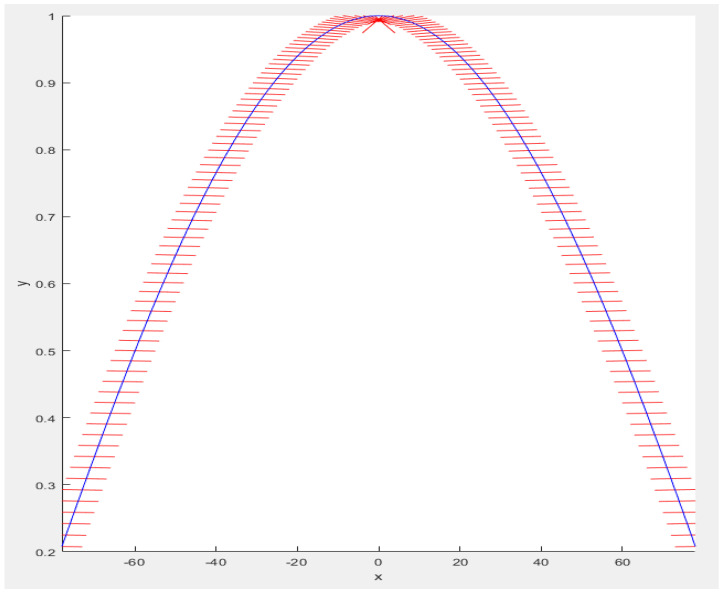
Direction of expected value formation for γ = π. The red lines represent the direction of the expected value formation, while the blue line represents an ideal cosine.

The value $x_{i}$ represents the x-value at which the line is initialized and cuts the cosine. The slope of the line is determined by γ. The two approaches specified previously correspond to γ→0 and γ→∞, being vertical, respectively horizontal lines. In Figure 4, Figure 5 and Figure 6, we represent the previous approaches as well as an example of straight lines with γ= π. 

The next step is calculating the pairs (*x*, *y*) and then translating them into pairs of $\left(\alpha_{assignment},\;\alpha_{diff}\right)$.

In [25], the author proposes a similar process that involves the use of the Nadaraya-Watson kernel in order to calculate all the (*x*, *y*) pairs and release the SACC. However, this approach is very memory-consuming in newer generations of radar due to the need to cover multiple chirp sequence—center frequency combinations; as such, a unique SACC is needed for each such combination, while also considering an iterative process of regression, as in [21]. 

Our proposed method calculates weighted average x-values around the presented straight lines m($x_{i},\;x)$. In order to achieve this, the normal component of the distance $d_{i,n}$ of a point ($x_{n}$, $y_{n}$) to the straight line m($x_{i},\;x)$ is calculated using the Hessian normal form [26]:

(19) $$d_{i,n}=\frac{s_{i}\cdot x_{n}-y_{n}-s_{i}\cdot x_{i}+\cos\left(x_{i}\right)\;}{\sqrt{s_{i}{}^{2}+1}}\;$$

It is important to note that only data points for which sign($x_{n}$) = sign($x_{i}$) can be used here. This is due to the fact that it is possible that a straight line intersects the cosine curve at a further point in addition to the initialization point, whereby targets can also be located on the straight line in the vicinity of the second intersection point, thereby showing why the low-angle area is very vulnerable.

Now it is sufficient to determine the x-value of the center of gravity, $x_{i,s}$. The calculation can be carried out adaptively for each point $x_{i}$ using a weighted average approach, considering the ego velocity, the SNR of the target, the distance of the point to the straight line defined by $x_{i}$ and the straight line defined by the next initialization point, $x_{i+1}$:

(20) $$x_{i,s}\left(k\right)=\frac{\left(x_{i,s}\left(k-1\right)\cdot G_{i}\left(k-1\right)+g_{i}\left(d_{i,k},v_{ego,k},\;SNR_{k}\right)\cdot x_{k}\;\right)}{G_{i}\left(k\right)}$$

(21) $$G_{i}\left(k\right)=G_{i}\left(k-1\right)+g_{i}\left(d_{i,k},v_{ego,k},\;SNR_{k}\right)\;$$

where $G_{i}\left(k\right)$ is the current total weight, $x_{i,s}\left(k\right)$ is the current weighted average at supporting point *k* and $g_{i}$ is a function that describes the weight for the current radar target used and it is calculated in the following way:

(22) $$g_{i}\left(d_{i,k},v_{ego,k},\;SNR_{k}\right)=g_{d}\;\cdot \;g_{vego}\;\cdot \;g_{SNR}$$

where $g_{d}$, $g_{vego}$ and $g_{SNR}$ are the weights based on distance, ego velocity and SNR.

(23) $$g_{d}=\left\{\begin{array}{l} e^{-\frac{d_{i}{}^{2}}{2\sigma_{i}{}^{2}}},\;d_{i}<d_{i,next}\\ 0,\;else \end{array}\right.$$

(24) $$g_{vego}=\left\{\begin{array}{c} linear\;interpolation\\ 1,\;v_{ego}>v_{max}\\ 0,\;v_{ego}<v_{min} \end{array}\right.$$

(25) $$SNR_{k}=\left\{\begin{array}{c} linear\;interpolation\\ 1,\;\mathrm{SNR}>SNR_{max}\\ 0,\;\mathrm{SNR}<{\mathrm{SNR}}_{min} \end{array}\right.$$

Knowing $x_{i,s}$, we can calculate $y_{i,s}$ in the following way, assuming $x_{i,s}$ already contains the mounting azimuth angle correction:

(26) $$y_{i,s}=\cos\left(x_{i,s}+\alpha_{diff}\;\right)$$

This will result in the estimation of the bias at the point $x_{i,s}$, thus providing the desired $\left(\alpha_{assignment},\;\alpha_{diff}\right)$ pair, where $\alpha_{assignment}=x_{i,s}$:

(27) $$\alpha_{diff}=sign\left(x_{i,s}\right)\cdot arccos\left(y_{i,s}\right)-x_{i,s}\;$$

### 3.2. Correction Validation

The correction $\alpha_{diff}$ and the assignment angle $\alpha_{assignment}$ will be transmitted as a pair to the IACC update block. As seen in Section 3, the IACC and SACC have a layout resembling a table. They have a number of points, a minimum distance between adjacent points, and a beginning angle, called $\alpha_{start}$.

The next step is to determine the index *i* of the assignment’s closest left neighbor, as shown in (28):

(28) $$i=\left[\;\frac{\alpha_{assignment}-\alpha_{start}}{\alpha_{step}}\right]$$

The linear least squares fitting method will be used to update the left and right side corrections connected to *i* and *i* + 1, providing a slope and an intercept based on $\alpha_{diff}$, $\alpha_{assignment}$ and a number of left and right neighboring pairs of (*α*, IACC(*α*)). As a result, the internal correction for the left neighbor will be as follows:

(29) $$\alpha_{diff}\left(i\right)=a\cdot \left(\alpha_{start}+i\cdot \alpha_{step}\right)+b$$

where *a* and *b* are the slope and the intercept, resulted from applying the least squares fitting method [24]. 

As shown in [21], the IACC is obtained by using an exponential moving average filter with factor *f* [22], described in (30):

(30) $$IACC_{current}\left(i\right)=IACC_{previous}\left(i\right)+f\cdot \left(\alpha_{diff}\left(i\right)-IACC_{previous}\left(i\right)\right)\;$$

The next step before updating the SACC is to apply a smoothing technique over the current IACC, producing a smoothed IACC. This is carried out after a number of *N* updates of the IACC have occurred, denoted as plausibility cycles. The smoothed correction for the supporting point *k* is derived based on the nearby corrections and their quality measures, as illustrated in (31). Our smoothing mechanism approach is based on employing a quality measure of the IACC updates of each supporting point as weights:

(31) $$SIACC\left(k\right)=\frac{\sum_{k-2}^{k+2}IACC\left(k\right)\frac{1}{\varepsilon_{Nk}}}{\sum_{k-2}^{k+2}\frac{1}{\varepsilon_{Nk}}}$$

where *N* is the number of updates for the supporting point in the current update cycle, SIACC (Smoothed IACC) is the resultant IACC, following the smoothing method, and $\varepsilon_{Nk}$ is the selected quality measure of the updates.

Compared to [21], where we have selected an exponential moving average-based standard error for the quality metric, we now propose an absolute difference between the simple average of the $\alpha_{diff}{}_{j}\left(k\right)$ values from the current plausibility cycle and IACC(*k*):

(32) $$\varepsilon_{Nk}=\left|\frac{\sum_{i=1}^{N}\alpha_{{diff}_{i}}\left(k\right)}{N}-IACC\left(k\right)\;\right|$$

**Figure 7:**
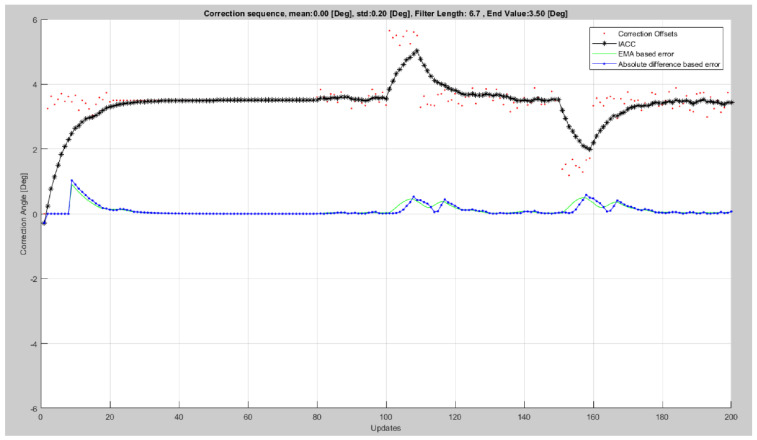
IACC correction sequence (black) based on calculated corrections (red), affected by white gaussian noise of null mean and standard deviation equal to 0.2°, and the resulting $\varepsilon_{Nk}$ for the mentioned methods (green—the previous method; blue—the current method).

The new error calculation method is improved, compared to the one proposed in [21], because it is a faster divergency measure, has a better adaptation time that is dependent on the exponential moving average filter factor, and is overall steadier, as seen in Figure 7, where we compare the two methods.

The previous SACC value will take the value of the new SIACC value for supporting point *k*. We suggest a recalculation based on the SACC neighbors, *k* − 1 and *k* + 1, in order to overcome the angular ambiguities that the SACC might introduce.

The updated SACC value for supporting point *k* is as follows:

(33) $$SACC\left(k\right)=\frac{SACC\left(k-1\right)+SACC\left(k+1\right)}{2}$$

The driving situations that arise during the course of a vehicle’s lifespan are as varied as the driving environment. However, the SACC behavior must also take into account the sensor’s aging and any potential situations in which the cover or bumper could be struck. It must also be accurate and simple to adjust over the duration of the sensor’s lifespan.

We suggest using a global coefficient of variance for this, which captures the SACC’s overall stability and robustness. First, as shown in (34), we determine the local variance using the most recent IACC updates:

(34) $$\sigma_{IACC\left(k\right)}{}^{2}=\frac{\sum_{j=1}^{N}{\left(\alpha_{{diff}_{j}}\left(i\right)\right)}^{2}-\frac{1}{N}{\left(\sum_{j=1}^{N}\alpha_{{diff}_{j}}\left(i\right)\right)}^{2}}{N-1}\;$$

The overall variance coefficient is calculated using an exponential moving average filter of factor $f_{\sigma^{2}}$:

(35) $$\sigma_{current}{}^{2}=\sigma_{previous}{}^{2}+f_{\sigma^{2}}\cdot \left(\sigma_{new}{}^{2}-\sigma_{previous}{}^{2}\right)$$

The absolute value of all smoothed differences (SIACC) between the measured azimuth and the references can be used to describe the convergence of the SACC. Based on an exponential moving average filter of factor $f_{\theta }$, the remaining offset is expressed in (36):

(36) $$\theta_{current}=\theta_{previous}+f_{\theta }\cdot \left(\theta_{new}-\theta_{previous}\right)\;$$

Finally, the SACC progress can be expressed as a function of θ, as shown in (37):

(37) $$\rho_{SACC}=\left\{\begin{array}{c} \frac{\theta_{min}}{\theta_{current}}\cdot 100\;\left[\%\right],\theta_{current}>\theta_{min}\\ 100\%,\;otherwise \end{array}\right.$$

where ρ is the current SACC progress. 

## 4. Numerical Results

We give simulation results for the proposed approaches in this section. A commercial millimeter wave automotive radar with the following specifications is used to test the algorithm: high resolution in distance, relative velocity, and angle; a center frequency of 76.5 GHz; azimuth and elevation angle fields of view of ±90° and ±15°, respectively; and an Ethernet communication interface. The conclusions are based on real-world data from several test drives conducted on city, highway, and country road types of streets under favorable climatic and driving conditions for a rear-left radar sensor. This information is utilized as input for our simulation environment. The simulation environment is software-in-the-loop (SIL)-based tooling that uses raw data as input and, based on it, performs resimulation of the entire radar chain. With this method, we can test the program code compiled for the target sensor without including real hardware, making it easier to test different parameter combinations. 

### 4.1. Vertical Misalignment 

**Figure 8:**
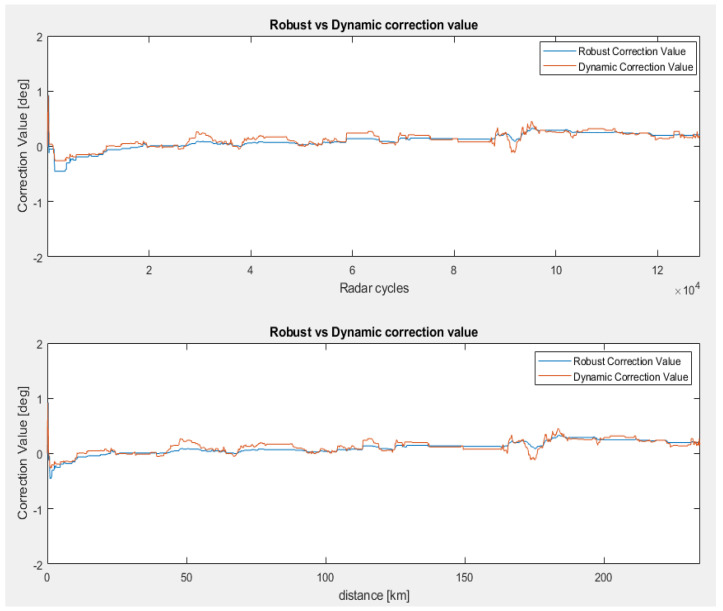
Vertical correction for the 0° scenario. Correction angle over radar cycles (**upper picture**) and correction angle with respect to the driven distance (**lower picture**) [22].

In the first scenario, we tested the radar sensor mounted on a bike rack so that the sensor is not affected by bumper influences and ensures an elevation mounting angle close to 0° using a goniometer. The results are presented in Figure 8, where the correction values are shown as a function of radar cycles and driven distance, respectively. We can observe a more dynamic behavior from both algorithms until the convergence point is reached, but this behavior is maintained by the dynamic correction method. The average correction value for both estimation techniques is centered around 0° (0.097° for robust filtering and 0.121° for dynamic filtering).

**Figure 9:**
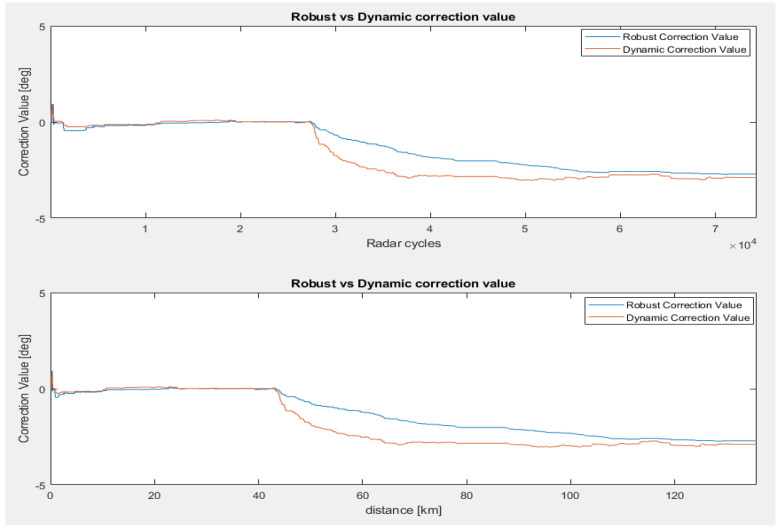
Vertical correction for the 3° error scenario (second scenario). Correction angle over radar cycles (**upper picture**) and correction angle with respect to the driven distance (**lower picture**) [22].

In the second misalignment scenario, we introduced an artificial error of 3° after approximately 40 km of driving and tested the convergence of both algorithms. Figure 9 compares the efficiency of dynamic filtering versus robust filtering in terms of the time and distance required to converge to the new correction value. The dynamic filtering method reaches the convergence point within 1° of accuracy, 20 km faster than the robust method (see Figure 9, lower picture).

**Figure 10:**
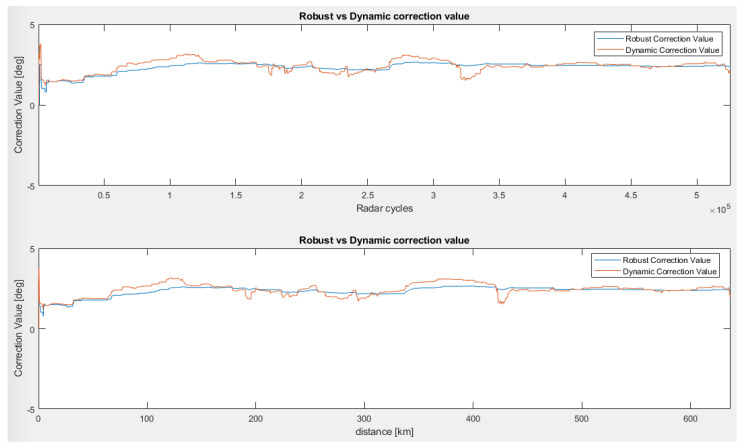
Vertical correction for a long-term evaluation (third scenario). Correction angle over radar cycles (**upper picture**) and correction angle with respect to the driven distance (**lower picture**) [22].

In the third scenario, we tested the sensor behind the bumper in a test drive for over 600 km in different environmental conditions in order to test the stability of the proposed algorithms. This scenario involves driving a vehicle on different types of roads (city, highway) and different weather conditions (snow/no snow). Figure 10 illustrates the stability of the vertical correction over a long period of time, and the statistics can be observed in Table 1.

In the fourth and last scenario, we tested the capabilities of the dynamic algorithm as an initial calibration method to demonstrate the possibility of achieving 0 km performance with the general purpose of ensuring an accurate starting angle before the vehicle leaves the OEM factory. For this, we executed seven tests, each consisting of straight-driving the car in a local validation area for a few hundred meters. 

**Figure 11:**
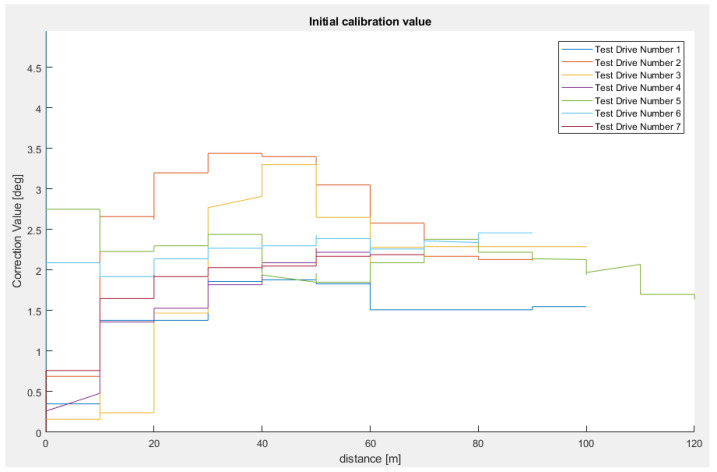
Initial vertical alignment scenario over distance (scenario 4). Correction values with respect to driven distance for seven test drives [22].

**Figure 12:**
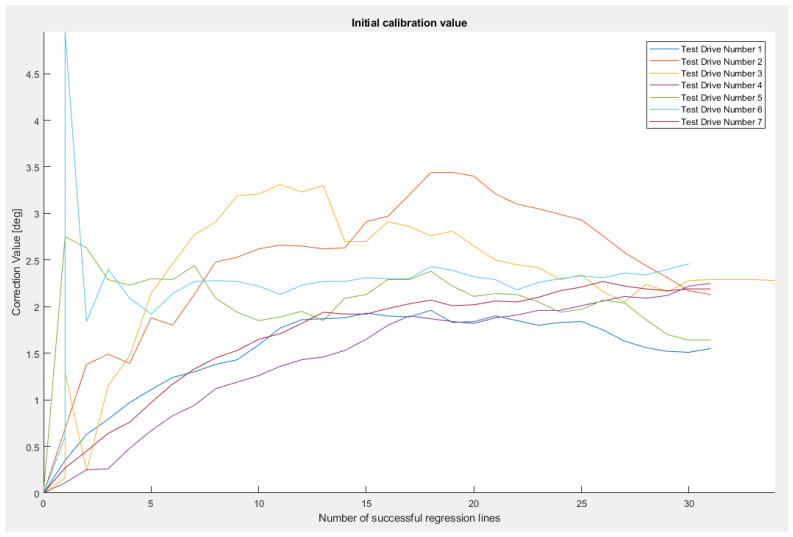
Initial vertical alignment scenario over the number of successful least squares regressions (scenario 4). Correction values with respect to the number of successful regression lines for seven test drives [22].

Figure 11 and Figure 12 present the evolution of the vertical correction angle for the tests, both over distance and over the number of successful least squares regression lines generated during each test. 

We can observe that most of the tests concluded in convergence in less than 100 m (with the exception of Test Drive Number 5, which finishes in 120 m) and needed only 30 successfully performed least squares regressions.

Using the values obtained at the end of each test, we obtained a mean vertical alignment angle of 2.071°, a variance of 0.117°, and a mean calibration distance of 90 m.

### 4.2. Azimuth Bumper Error Compensation 

The results are based on real data from a test drive effectuated on a highway in optimal driving and environmental conditions for the rear left sensor. This data is used as input for our simulation environment. In the first scenario, we compare the performance of the previously used method for correction calculation based on road edge [21] with our new proposal.

**Figure 13:**
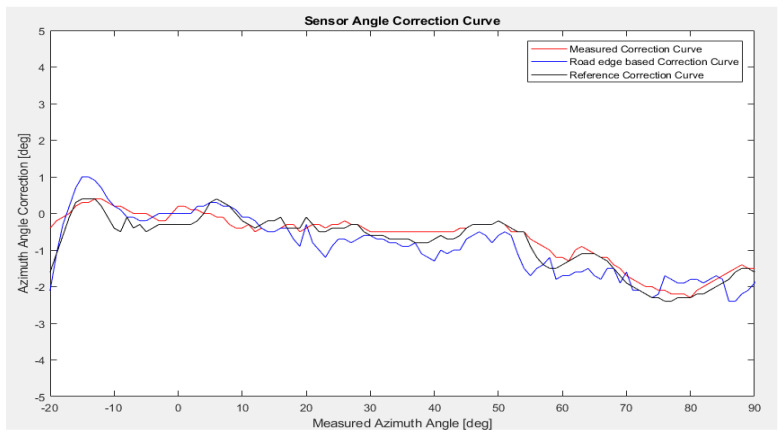
Comparison of the measured SACC against the Road-Edge-Based SACC and the reference SACC. Azimuth angle correction is shown with respect to the measured azimuth angle.

**Figure 14:**
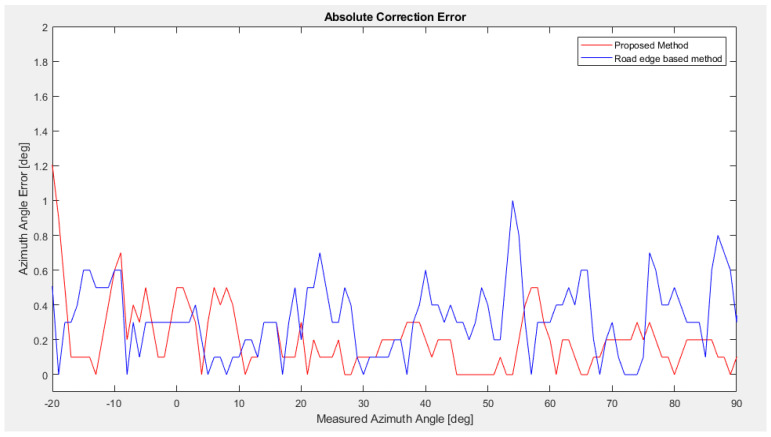
The absolute SACC error is shown with respect to the measured azimuth angle.

Figure 13 illustrates the calculated SACC for the rear left sensor, compared to the road edge-based approach, and the reference SACC values for the relevant angular interval. Figure 14 shows the SACC absolute error between the two methods and the reference SACC, excluding the two extremities at [−34°, 20°] and [90°, 126°], due to the angular errors, containing both bumper errors and an additional error based on the sensor limitation at the edge of the field of view that is outside of the scope of the method. The additional errors are caused by antenna coupling effects that lead to high angular distortion, in particular at the edge of the field of view. These errors are also highly sensitive to production tolerances. To reduce this sensor-related effect, a sensor-individual phase calibration at a certain number of reference angles is carried out within the end-of-line calibration (15 points in our case, denser at the edge of the field of view). By means of this phase calibration, a sensor-individual phase correction takes place, which reduces the angular distortions, especially at the edges of the field of view. The remaining angular distortions are then mainly caused by bumper effects. Moreover, this sensor limitation further explains the constraint of the method due to the lack of suitable radar raw targets, causing an error in the stationarity of the targets where stationary targets are being recorded as moving targets, thus being excluded by the algorithm. Further examples will be based on the rear sensor, mounted behind the bumper, that is affected only by bumper-related errors. 

We can observe that our proposed method has an overall better accuracy outside the [−10°, 10°] interval, which can be accounted for by the lack of usage of the elevation angle in the reference angle calculation, where at lower azimuth angles it can have a significant importance. Our new proposal performs better than our previous method, with a lower average absolute error (0.20° compared to 0.32°), excluding the two extremities at [−34°, 20°] and [90°, 126°] intervals. 

**Figure 15:**
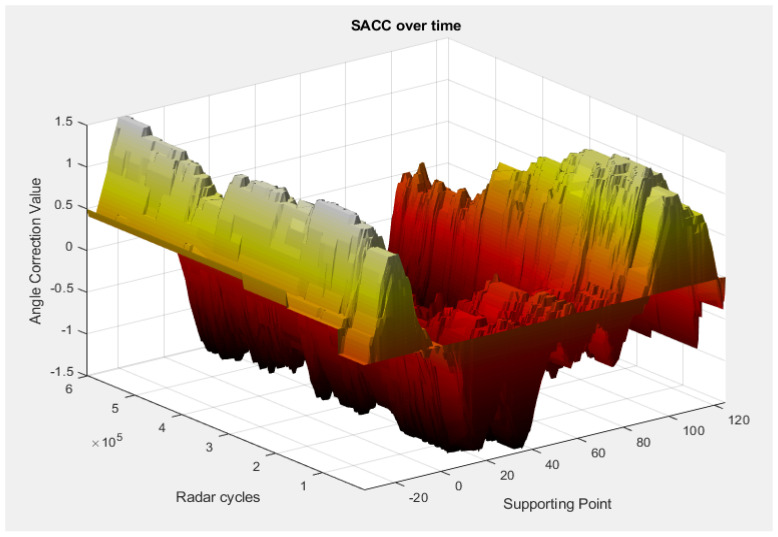
SACC values over time. The angle correction value is shown in a 3D representation with respect to radar cycles and supporting points.

**Figure 16:**
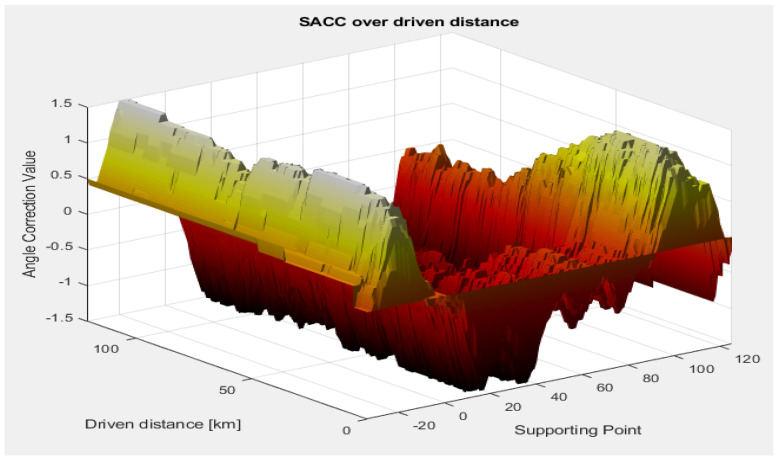
SACC values over driven distance. The angle correction value is shown in a 3D representation with respect to driven distance and supporting points.

In the next scenario, we tested the sensor behind the bumper, without the antenna coupling-related errors, in a test drive for over 100 km, in which the activation conditions were met in different environmental conditions, in order to test the stability of the algorithm. This scenario involves driving a vehicle on different types of roads (city and highway) and different weather conditions (snow/no snow). Figure 15 and Figure 16 illustrate the shape of the SACC over time and over the driving distance.

**Figure 17:**
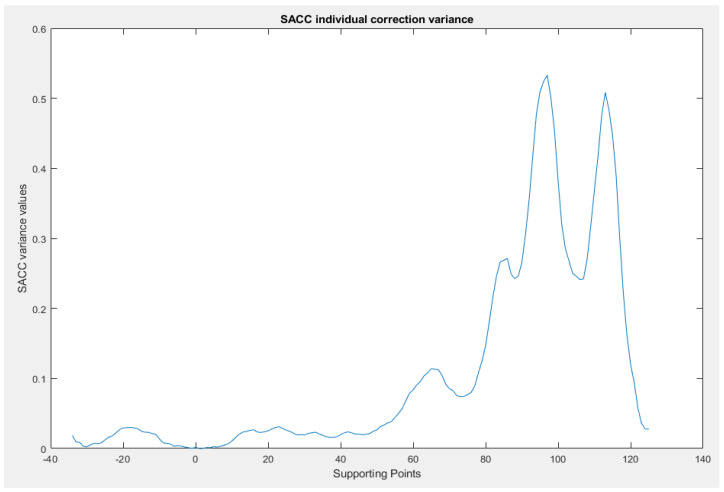
SACC variance for each individual supporting point. The SACC variance is shown with respect to the supporting points.

The SACC demonstrates a fast convergence, being able to converge in less than 10 SACC events (Figure 15, the Radar Cycles axis), or approximately 10 driven kilometers (Figure 16, the Driven Distance axis), remaining in a stable state with the slight exception of the extremities, as proven by the individual supporting point variance, presented in Figure 17, where we can observe a variance lower than 0.1° for all the supporting points with the exception of the edge of the field of view. 

## 5. Discussion

### 5.1. Discussion on Vertical Misalignment

The elevation mounting angle compensation method proposed is, to the best of our knowledge, the only vertical alignment calculation method that is tailored specifically for elevation mounting angle error compensation. All studied papers contain either azimuth-only compensation or joint azimuth-elevation calculations. Having a joint calculation is arguably better in the normal operation mode of a radar sensor, but having different algorithms based on different methods, besides the accuracy shown in our paper, can be beneficial in case a system failure happens in such a way that the inputs necessary for the joint azimuth-elevation calibration are not valid anymore, the impact being a false or lack of calculation for both compensation values.

Secondly, we proposed the usage of two sets of values based on a robust and dynamic filtering of the calculated compensation values.

The main disadvantage of the concept is that the method is not suitable for the side sensors, as preferably the best targets for the algorithm would be the ones around stationary longitudinal objects like road edges (metal guardrails from the highway). For side sensors, these would actually indicate a roll angle misalignment. For elevation compensation in lateral sensors, the height bins would have to be selected in the latitudinal position, not the longitudinal position, and the biggest issue is that latitudinal bins would contain different kinds of environments that would not be homogenous. One latitudinal bin might include targets from the road edge; the next bin would include targets from concrete or trees; and the next one from houses, meaning the linear regression applied over the heights of the bins would not provide an accurate compensation value.

In regard to the computational time and complexity of the method, the process is not very extensive due to the usage of the average height of the longitudinal bins as the input for the linear regression instead of directly using the accumulation of all suitable targets, meaning that for one occurrence of a successful linear regression (one calibration event), the time complexity is O($nBins^{3}$), where $nBins\geq \;Min_{Bins}$ [27]. Considering the entire process, it is very difficult to express the time complexity in terms of the O notation due to the multitude of processes that take place, from the target selection for the average bin height calculation, which is highly dependent on the environment in which the vehicle is driving, to the longitudinal bin height calculation and eventually the regression calculation. In terms of the time it takes for the algorithm to converge, it is also a process that includes the environment and the given values for the exponential moving average filtering factors, as well as the desired $Min_{Bins}$ and the performance of the radar sensor used in terms of the maximum number of targets that it can detect. In our study, we found that in good environmental conditions (mainly highway driving, where there is a road edge on both sides of the vehicle), the algorithm converges in less than 10 km and keeps calculating a steady value after the convergence takes place. Moreover, in the context of an initial online calibration in the manufacturer’s plant or service, given more relaxed parameters and accepting a lower accuracy, as it is usually expected by automotive OEMs, the method achieved convergence in a local validation area that resembles a factory environment with an average driven distance of 90 m. 

### 5.2. Discussion on Azimuth Bumper Error Compensation 

The proposed bumper compensation method is better than the previously presented one as it does not depend on the usage of stationary references like the road edge, consequently using more radar targets, taking a lower time to converge, and providing an initial SACC.

The main disadvantage of the method consists mainly of its dependency on the corrected ego velocity, as the velocity that originates from the vehicle’s speedometer is not accurate enough due to odometry errors caused by a lot of issues (e.g., deflated tires). An error in velocity will negatively influence the calculation of the reference azimuth angle, as seen in Equations (11)–(17).

In regard to the computational time and complexity of the method, we have the same remarks as in Section 5.1 due to employing a target selection phase that takes place in every radar cycle. The SACC’s creation is also a process that takes place over multiple radar cycles. While a SACC update is released only after *N* radar cycles pass, in which there is at least one suitable target, the IACC is updated constantly in every radar cycle for all suitable targets found, meaning a least squares regression with a number of points from the right and left sides of the current supporting point is used, having a similar time complexity as in Section 5.1. Moreover, given that the overall process is dependent on the environment and the sensor performance, the parameters in usage (e.g., the number of plausibilization cycles, exponential moving average factor *f*) can be used to fine tune the method, resulting in obtaining the accuracy and convergence time desired by the OEMs according to the requirements and sensor capabilities. In our study, we have found that a big improvement is being made even by the first SACC event, but the convergence of the SACC is complete after only 10 such events, or approximately 10 km.

## 6. Conclusions

A new unsupervised online calibration approach for calculating the vertical misalignment of an automotive radar is proposed in this paper. The method is based on stationary targets and employs two automatic calibration values based on two sets of values for the parameters, such that one value is stable and accurate in long-term driving scenarios, while the other value provides faster misalignment detection in the event of an accident. Based on a hysteresis procedure between the dynamic and robust values, the correction value to be used is decided. The method presents an accurate mean angle of 0.097° in a scenario where the sensor was not misaligned, as well as detecting a 3° misalignment in less than 20 km.

Further, an improvement to an existing autocalibration method was introduced for performing a continuously adjustable and tunable azimuth automatic calibration process that can adapt to any vehicle, accurately compensate azimuth local offsets in a robust manner in the [−20, 80]-degree interval, and provide statistical data that characterize the resulting angle correction curve. The novelty of the method consists of the fact that it is based on stationary targets but is not dependent on any stationary structures like road edges, considerably increasing the usage and update rate of the algorithm. The method converges in less than 10 km, with most supporting points registering a variance of less than 0.1°.

The algorithms have been evaluated in a software-in-the-loop (SiL) environment and yielded good results as an initial and continuous calibration. 

Future work will include the investigation of various regression and filtering approaches as well as the application of the concept to the calculation of the roll angle of side sensors for unsupervised vertical misalignment detection. For the azimuth angle bumper error compensation approach, future work includes the study of different regression approaches as well as the extension of the method in the elevation direction.

## Figures and Tables

**Table 1:** Comparison of the resulting correction values for scenario 3.

Correction Method	Mean Value [°]	Variance [°]
$\mathrm{Robust}\;\mathrm{filtering}\;\Delta \alpha_{r}$	2.323	0.103
$\mathrm{Dynamic}\;\mathrm{filtering}\;\Delta \alpha_{d}$	2.400	0.159

## Data Availability

The data presented in this study are available on request from the corresponding author. The data is not publicly available due to privacy concerns.
